# Age-stratified reference ranges for adenoid hypertrophy in children: a single-center retrospective study

**DOI:** 10.3389/fped.2025.1639498

**Published:** 2025-08-14

**Authors:** Enfu Tao, Wei Liang, Hongdan Gu, Junfen Zhou, Changhua Zheng, Junhui Yuan

**Affiliations:** ^1^ Department of Neonatology and Neonatal Intensive Care Unit, Wenling Maternal and Child Health Care Hospital, Wenling, Zhejiang, China; ^2^ Department of Radiology, Wenling Maternal and Child Health Care Hospital, Wenling, Zhejiang, China; ^3^ Department of Pediatrics, Wenling Maternal and Child Health Care Hospital, Wenling, Zhejiang, China

**Keywords:** adenoid hypertrophy, *A*/*N* ratio, adenoid depth, nasopharyngeal depth, nasopharyngeal radiography

## Abstract

**Introduction:**

Adenoid hypertrophy (AH) is prevalent in 35%–70% of the global pediatric population, leading to airway obstruction and sleep disturbances. Current diagnostic criteria for the adenoid-to-nasopharyngeal (*A*/*N*) ratio lack age-specific adjustments, potentially resulting in diagnostic inaccuracies.

**Methods:**

This retrospective study assessed pediatric outpatients aged 1–12 years who underwent lateral nasopharyngeal radiography. Measurements of adenoid depth (AD), nasopharyngeal depth (ND), and *A*/*N* ratios were recorded, and age-stratified percentiles (P5-P95) were calculated for four distinct age cohorts. The relationships between AD, ND, and *A*/*N* ratios and age were analyzed. Measurements were conducted by two independent radiologists, with any discrepancies adjudicated by a senior expert.

**Results:**

In this investigation involving 2,629 outpatient children aged between 1 and 12 years, the median AD remained consistent at 14–15 mm, whereas ND increased from 21 to 27 mm, resulting in a decrease in the *A*/*N* ratio from 0.68 to 0.56. Pathological hypertrophy was identified in 42% of children aged 1–3 years, compared to 13.7% in those aged 10–12 years, with no significant sex-based differences observed. Age-specific reference ranges showed that both AD and ND increased with age, whereas the *A*/*N* ratio decreased. A positive correlation was found between AD and both ND and the *A*/*N* ratio, while ND exhibited a negative correlation with the *A*/*N* ratio. Significant discrepancies were noted between age-specific *A*/*N* ratio percentiles and the current fixed diagnostic criteria for children aged 1–12 years. The study established percentile-based reference values (P5–P95) for AD, ND, and the *A*/*N* ratio across four pediatric age groups.

**Conclusions:**

This study established percentile-based reference values (P5–P95) for AD, ND, and the *A*/*N* ratio across four pediatric age groups, thereby recommending age-specific diagnostic thresholds for AH in clinical settings.

## 1 Introduction

Adenoid hypertrophy (AH) is a prevalent condition among pediatric populations, with prevalence rates estimated to be between 35% and 70% globally (1). It is a significant contributor to upper airway obstruction, leading to chronic nasal congestion, snoring, sleep-disordered breathing, and, in severe cases, cognitive impairment (2–4). Accurate evaluation of nasopharyngeal airway dimensions and adenoid size is critical for differentiating between physiological growth and pathological hypertrophy; however, standardized normative values exhibit considerable variability across different age groups and geographic regions.

In the diagnosis of AH, the adenoid-to-nasopharynx (*A*/*N*) ratio is a crucial imaging parameter for evaluating adenoid size and the extent of hypertrophy (5). Although previous studies have affirmed the reliability and clinical applicability of this measurement (6), they exhibit several limitations that warrant further investigation. For instance, the study by Acar et al. established the *A*/*N* ratio as a “useful, tolerable, and confident diagnostic method” for pediatric patients (6), but it was limited by a relatively small sample size (*n* = 46). In contrast, a comprehensive analysis by Guo et al. investigated 1,188 children aged 8 months to 13 years, utilizing machine learning techniques to analyze lateral nasopharyngeal radiographs. Their findings indicated an accuracy comparable to that of radiologist evaluations (5). Nonetheless, this study was restricted to treated cases, potentially introducing selection bias by omitting symptomatic children who did not require intervention. Furthermore, neither this study nor the preceding one provided detailed age-stratified analyses of nasopharyngeal dimensional changes. Additionally, the diagnostic thresholds for the *A*/*N* ratio exhibit considerable variability across different studies. Some research designatesan *A*/*N* ratio of ≤0.60 as normal, a ratio of 0.61–0.70 as indicative of moderate hypertrophy, and a ratio of ≥0.71 as suggestive of pathologic hypertrophy (7). Conversely, other studies classify a ratio of 0.61–0.70 as indicative of hypertrophy (8). This discrepancy highlights the lack of consensus and emphasizes the necessity for standardized, age-specific diagnostic criteria.

To address the limitations of previous research, our large-scale retrospective study analyzed 2,629 pediatric outpatients who underwent lateral nasopharyngeal radiography, thereby providing significantly enhanced statistical power and clinical generalizability compared to earlier investigations. This extensive dataset facilitates the establishment of precise, age-stratified reference ranges (1–3, 4–6, 7–9, and 10–12 years) for both *A*/*N* ratios and nasopharyngeal dimensions within a real-world outpatient population.

## 2 Materials and methods

### 2.1 Study design and ethical considerations

This single-center retrospective observational study was conducted in accordance with the STROBE guidelines and approved by the Institutional Review Board of Medical Ethics Committee of Wenling Maternal and Child Health Care Hospital (2025-IRB-004), and written informed consent was obtained from the patient's parents.

### 2.2 Study population

We conducted a screening of all pediatric patients (aged <18 years) who underwent nasopharyngeal lateral radiography at the outpatient clinic of our institution from January 21, 2019, to April 30, 2025. The patients were categorized into seven age-based subgroups: <1 year, 1–3 years, 4–6 years, 7–9 years, 10–12 years, 13–15 years, and 16–18 years. The exclusion criteria were as follows: (1) congenital nasopharyngeal anomalies (e.g., choanal atresia, craniofacial syndromes), (2) history of nasopharyngeal surgery or radiotherapy, (3) imaging related to acute trauma, and (4) radiographs of insufficient quality for accurate measurement.

### 2.3 Image collection and radiographic analysis

**Figure 1:**
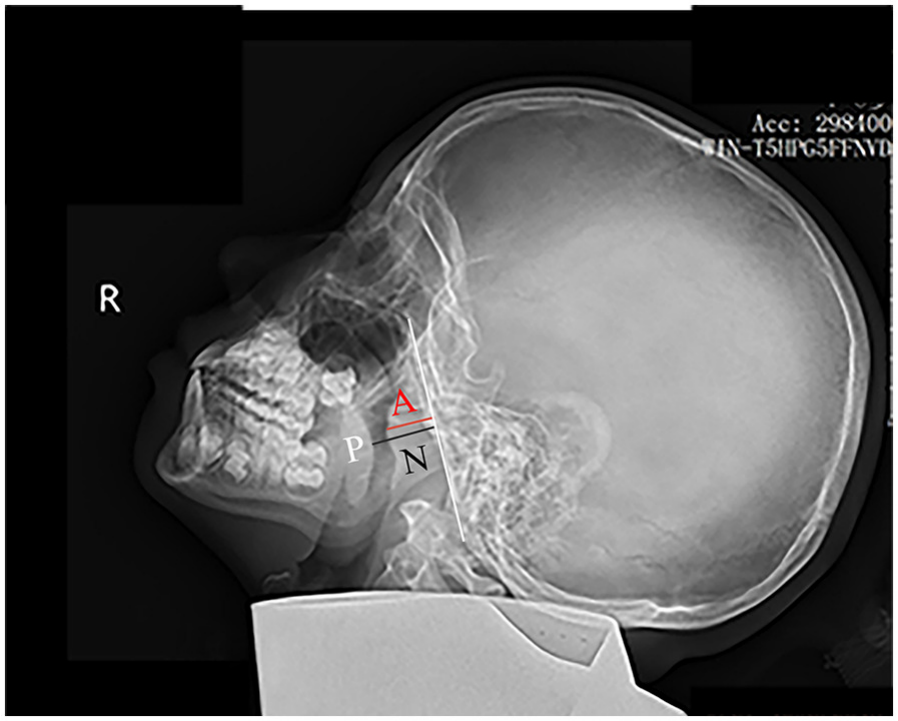
Measurement of the *A*/*N* ratio on a lateral nasopharyngeal x-ray. The white line marks the straight portion of the basiocciput's anterior margin. The adenoid depth (AD) is measured as the perpendicular distance from this line to the point of maximal adenoid prominence (red line). The nasopharyngeal depth (ND) extends from the posterior hard palate (P) to the anteroinferior edge of the sphenobasioccipital synchondrosis (black line).

Children were positioned laterally in a standing posture and instructed to breathe calmly during the imaging procedure. Digital x-ray images were obtained using x-ray machines (HF50-RA, Beijing Wandong Medical Technology Co., Ltd., China). Lateral nasopharyngeal radiographs were captured with a tube current of 100–125 mA, a tube voltage of 60–70 kV, and a filming distance of 180 cm. Two board-certified pediatric radiologists, blinded to all clinical information, independently performed all measurements. In instances of discrepancy, a senior radiologist with over 20 years of expertise in imaging diagnosis was consulted. The measurements of AD, defined as the thickness of the nasopharyngeal roof and the soft tissue of the posterior wall, as well as the nasopharyngeal diameter ND and the *A*/*N* ratio, were calculated in accordance with established methodologies (5, 9). Figure 1 delineates the measurement techniques for both the AD and ND, as previously described (5).

### 2.4 Adenoid hypertrophy imaging criteria

The criteria established for AH indicate that an *A*/*N* ratio of ≤0.60 is classified as normal; a ratio of 0.61–0.70 is categorized as moderate hypertrophy; and a ratio of ≥0.71 is considered indicative of pathological hypertrophy (7).

### 2.5 Statistical analysis

All analyses were conducted using SPSS version 26.0 (IBM Corp). Continuous variables were evaluated for normality through Shapiro–Wilk tests and *Q*–*Q* plots. Normally distributed data are presented as mean ± standard deviation (SD), while non-normally distributed data are reported as median (minimum-maximum). Categorical variables are summarized as frequencies and percentages. Group comparisons were performed using the following methods: (1) for continuous variables, independent *t*-tests or ANOVA (with Tukey's *post-hoc* analysis) were utilized for normally distributed data, while Mann–Whitney *U* tests or Kruskal–Wallis tests (with Dunn's *post-hoc* analysis) were applied for non-normally distributed data; (2) for categorical variables, chi-square tests or Fisher's exact tests were employed. Statistical correlations were assessed using Pearson's correlation coefficient for normally distributed continuous variables and Spearman's rank correlation coefficient for non-normally distributed or ordinal data. To evaluate the consistency between the two board-certified pediatric radiologists who performed all measurements, both simple agreement percentages and Cohen's kappa coefficients were calculated. Simple agreement was calculated as the percentage of cases where both raters' measurements fell within the same clinical classification category for AH (normal: ≤0.60; moderate: 0.61–0.70; pathological: ≥0.71). Kappa values were used to determine inter-observer agreement, with values interpreted as follows: 0–0.20 indicated very low consistency, 0.21–0.40, fair consistency, 0.41–0.60, moderate consistency, 0.61–0.80, high consistency, and 0.81–1.00, perfect consistency (10). A *P*-value of less than 0.05 was considered statistically significant.

## 3 Results

### 3.1 Subject enrollment and exclusion process

**Figure 2:**
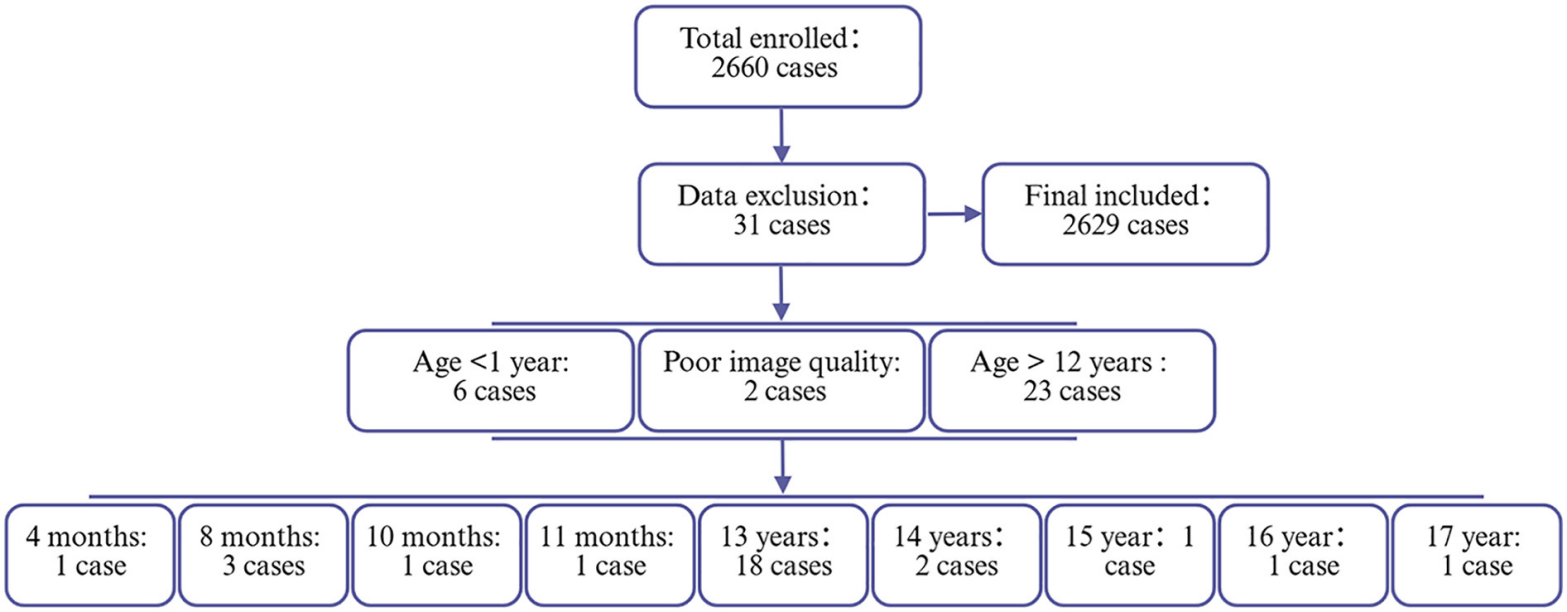
Flowchart of participant enrollment and exclusion criteria. This flowchart illustrates the screening process of the study population. A total of 2,660 cases were initially enrolled. After applying exclusion criteria (*n* = 31), 2,629 cases were included in the final analysis.

Between January 21, 2019, and April 30, 2025, a total of 2,660 pediatric outpatients underwent nasopharyngeal lateral radiography. The ages of these patients ranged from 1 year to 17 years. However, six cases were excluded due to the patients being under 1 year of age, an additional 23 cases were excluded for exceeding the age of 12 years, and two cases were excluded due to suboptimal imaging quality. Consequently, 2,629 cases were included in the final analysis. The study population was categorized into four age groups for analysis: 1–3 years, 4–6 years, 7–9 years, and 10–12 years. The enrollment and exclusion process for subjects is depicted in Figure 2.

### 3.2 Characteristics of the study population

The study ultimately included 2,629 pediatric patients, who were stratified into four distinct age groups: 1–3 years (23.1%, *n* = 607), 4–6 years (45.1%, *n* = 1,186), 7–9 years (23.8%, *n* = 625), and 10–12 years (8.0%, *n* = 211). The distribution of participants across these age groups was found to be statistically significant (*P* < 0.001). Gender distribution was balanced, with no significant differences observed among the age groups (male: 42.0–44.8%; female: 55.2–60.2%; *P* = 0.57).

### 3.3 Adenoid depth, nasopharyngeal depth measurements and *A*/*N* ratio

Table 1 presents the distribution of AD, ND measurements, and the *A*/*N* ratio across various age groups. The AD values exhibited significant variability among the age groups (*P* < 0.001), with median values ranging from 14 mm (3, 22) in the 1–3 years group to 15 mm (3, 24) in the 4–6 years group, 15 mm (3, 26) in the 7–9 years group, and 15 mm (7, 24) in the 10–12 years group. *post-hoc* analyses indicated significant increases in AD when comparing the 1–3 years group to all older age groups (all *P* < 0.001), while no significant differences were detected among the older age groups (*P* > 0.05). Similarly, ND demonstrated a progressive increase with age (*P* < 0.001), with median values increasing from 21 mm (12, 32) in the 1–3 years group to 22.85 mm (13, 32) in the 4–6 years group, 25 mm (14, 33) in the 7–9 years group, and 27 mm (17, 34) in the 10–12 years group. *post-hoc* analyses confirmed statistically significant progressive increases in ND not only between the 1–3 years group and all older age groups but also among the older age groups themselves (all *P* < 0.001). Conversely, the *A*/*N* ratio decreased with advancing age: 0.68 (0.11, 0.95) in the 1–3 years group, 0.67 (0.17, 0.94) in the 4–6 years group, 0.61 (0.16, 0.93) in the 7–9 years group, and 0.56 (0.26, 0.88) in the 10–12 years group (*P* < 0.001). *post-hoc* analyses revealed significant decreases in *A*/*N* ratio across various age groups, specifically between the 1–3 year and the older groups (*P* < 0.01 for the 4–6 years group; *P* < 0.001 for all other comparisons).

**Table 1:** Baseline characteristics of the study population stratified by age groups (*n* = 2,629).

Characteristics	1–3 (years)	4–6 (years)	7–9 (years)	10–12 (years)	*P*-value
*n* (%)	607 (23.1)	1,186 (45.1)***	625 (23.8)^&&&^	211 (8.0)***^,^^&&&,###^	<0.001
Male	255 (42.0)	516 (43.5)	280 (44.8)	84 (39.8)	0.57
Female	352 (58.0)	670 (56.5)	345 (55.2)	127 (60.2)	
Adenoid depth (mm)	14 (3, 22)	15 (3, 24)***	15 (3, 26)***	15 (7, 24)***	<0.001
Nasopharyngeal depth (mm)	21 (12, 32)	22.85 (13, 32)***	25 (14, 33)***^,^^&&&^	27 (17, 34)***^,^^&&&,###^	<0.001
*A*/*N* ratio	0.68 (0.11, 0.95)	0.67 (0.17, 0.94)**	0.61 (0.16, 0.93)***^,^^&&&^	0.56 (0.26, 0.88)***^,^^&&&,##^	<0.001
Hypertrophy classification					<0.001
Normal (≤0.60)	125 (20.6%)	303 (25.5%)	286 (45.8%)***^,&&&^	127 (60.2%)***^,&&&,#^	
Moderate (0.61–0.70)	227 (37.4%)	429 (36.2%)	218 (34.9%)	55 (26.1%)	
Pathological (≥0.71)	255 (42.0%)	454 (38.3%)	121 (19.4%)***^,&&&^	29 (13.7%) ^&,#^	

Statistical significance was marked by symbols: Single for *P* < 0.05 (*, &, #), double for *P* < 0.01 (**, &&, ##), and triple for *P* < 0.001 (***, &&&, ###), comparing against 1–3, 4–6, and 7–9 years, respectively.

### 3.4 Adenoid hypertrophy

The classification analysis of AH across various age groups revealed significant developmental changes (*P* < 0.001) as presented in Table 1. The proportion of children with normal adenoids (*A*/*N* ratio ≤0.60) increased progressively in an age-dependent manner, rising from 20.6% in the 1–3 years age group to 60.2% in the 10–12 years age group. Intergroup comparisons indicated statistical significance (*P* < 0.001 for comparisons between 1–3 vs. 7–9 and 10–12 years, as well as 4–6 vs. 7–9 and 10–12 years; *P* < 0.05 for 7–9 vs. 10–12 years). Conversely, the prevalence of pathological hypertrophy (*A*/*N* ratio ≥0.71) showed a notable decline with increasing age, decreasing from 42.0% in the 1–3 years group to 13.7% in the 10–12 years group. These intergroup comparisons were statistically significant (*P* < 0.001 for comparisons between 1–3 and 4–6 vs. 7–9 years; *P* < 0.05 for comparisons between 10–12 vs. 4–6 and 7–9 years). The prevalence of moderate hypertrophy (*A*/*N* ratio 0.61–0.70) remained relatively stable, ranging from 34.9% to 37.4% among ages 1–9, before decreasing to 26.1% in the oldest age group; however, these variations did not reach statistical significance (all *P* > 0.05).

### 3.5 Sex-specific analysis of adenoid depth, nasopharyngeal depth measurements, *A*/*N* ratio and adenoid hypertrophy

The analysis of AD, ND, *A*/*N* ratio, and AH stratified by sex and age groups (*n* = 2,629) revealed no statistically significant differences (Table 2). The median AD measurements were comparable between males (15 mm; range 3–26) and females (14 mm; range 3–24) (*P* = 0.72), with similar distributions observed across all age groups. ND measurements also showed no significant sex differences, with males exhibiting a mean of 23 mm (range 12–34) and females a mean of 23 mm (range 13–33) (*P* = 0.81). The *A*/*N* ratio demonstrated a decline associated with age in both sexes, with values of 0.68 for both males and females aged 1–3 years, and 0.57 for males and 0.55 for females aged 10–12 years (overall *P* = 0.44). Classification of hypertrophy revealed comparable proportions between sexes: normal (≤ 0.60) cases comprised 58% of males *(P* = 0.81), moderate (0.61–0.70) cases comprised 56.2% of males (*P* = 0.40), and pathological (≥ 0.71) cases comprised 56.3% of males (*P* = 0.49), with consistent patterns observed across age groups.

**Table 2:** Sex differences of the study population stratified by age groups (*n* = 2,629).

Characteristics	Sex	All	1–3	4–6	7–9	10–12	*P*-value
Adenoid depth (mm)	Male	15 (3, 26)	14 (3, 21)	15 (3, 23)	15 (5, 26)	15 (7, 23)	0.72
	Female	14 (3, 24)	13 (3, 22)	15 (7, 24)	15 (3, 23)	15 (8, 24)	
Nasopharyngeal depth (mm)	Male	23 (12, 34)	21 (12, 27)	23 (13, 32)	25 (14, 33)	26 (17, 34)	0.81
	Female	23 (13, 33)	20 (13, 32)	22 (15, 30)	25 (16, 33)	27.5 (21, 33)	
*A*/*N* ratio	Male	0.64 (0.17, 0.94)	0.68 (0.13, 0.92)	0.65 (0.17, 0.94)	0.61 (0.17, 0.93)	0.57 (0.26, 0.88)	0.44
	Female	0.65 (0.11, 0.95)	0.68 (0.11, 0.95)	0.67 (0.35, 0.93)	0.61 (0.16, 0.90)	0.55 (0.30, 0.88)	
Hypertrophy classification	Male	1,494 (56.8)	352 (58)	670 (56.5)	345 (55)	127 (60.2)	0.70
	Female	1,135 (43.2)	255 (42)	516 (43.5)	280 (45)	84 (39.8)	
Normal (≤0.60)	Male	488 (58)	70 (56)	182 (60.1)	162 (56.6)	74 (58.3)	0.81
	Female	353 (42)	55 (44)	121 (39.9)	124 (43.4)	53 (417)	
Moderate (0.61–0.70)	Male	522 (56.2)	133 (58.6)	242 (56.4)	113 (51.8)	34 (61.8)	0.40
	Female	407 (43.8)	94 (41.4)	187 (43.6)	105 (48.2)	21 (38.2)	
Pathological (≥0.71)	Male	484 (56.3)	149 (58.4)	246 (54.2)	70 (57.9)	19 (65.5)	0.49
	Female	375 (43.7)	106 (41.6)	208 (45.8)	51 (42.1)	10 (34.5)	

*A*/*N*, adenoid-to-nasopharyngeal.

### 3.6 Age-specific reference ranges for adenoid and nasopharyngeal parameters

The percentile-based reference ranges (P5–P95) for adenoid and nasopharyngeal measurements were established across four pediatric age groups (Table 3). The reference intervals for AD were as follows: 9.0–18.0 mm for ages 1–3 years, 10.0–20.0 mm for ages 4–6 years, 9.0–20.0 mm for ages 7–9 years, and 8.6–21.0 mm for ages 10–12 years. The reference values for the *A*/*N* ratio exhibited an age-dependent decline, ranging from 0.48 to 0.84 for children aged 1–3 years, and decreasing to 0.35–0.76 for those aged 10–12 years.

**Table 3:** Distribution of adenoid depth, nasopharyngeal depth, and *A*/*N* ratio by age groups: percentile values (P5–P95).

Metric	Age group (years)	*n*	P5	P10	P25	P50	P75	P90	P95
Adenoid depth (mm)	1–3	607	9.0	10.0	12.0	14.0	16.0	18.0	18.0
	4–6	1,186	10.0	11.0	13.0	15.0	17.0	19.0	20.0
	7–9	625	9.0	11.0	13.0	15.0	17.0	19.0	20.0
	10–12	211	8.6	10.0	12.0	15.0	17.0	20.0	21.0
Nasopharyngeal depth (mm)	1–3	607	16.0	17.0	19.0	21.0	22.0	24.0	24.0
	4–6	1,186	18.0	19.0	21.0	22.9	24.0	26.0	26.0
	7–9	625	20.0	21.0	23.0	25.0	27.0	28.0	30.0
	10–12	211	21.6	23.0	25.0	27.0	29.0	31.0	32.0
*A*/*N* ratio	1–3	607	0.48	0.52	0.62	0.68	0.75	0.82	0.84
	4–6	1,186	0.45	0.50	0.60	0.67	0.74	0.79	0.83
	7–9	625	0.42	0.45	0.52	0.61	0.68	0.75	0.79
	10–12	211	0.35	0.38	0.46	0.56	0.65	0.72	0.76

*A*/*N*, adenoid-to-nasopharyngeal.

### 3.7 Relationship between age and adenoid depth, nasopharyngeal depth measurements, and *A*/*N* ratio

#### 3.7.1 Age vs. adenoid depth

**Figure 3:**
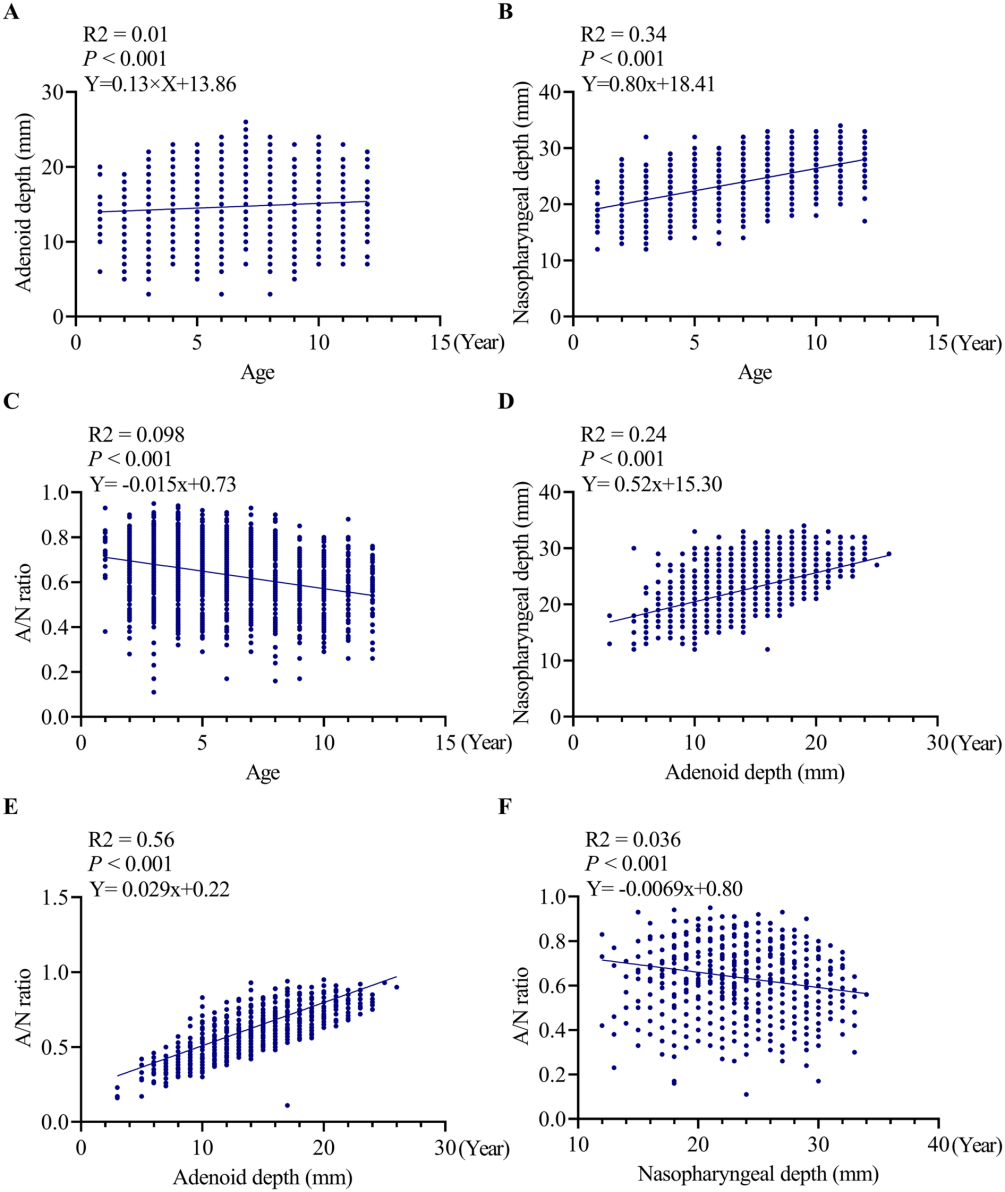
Correlation analyses between age, adenoid depth, nasopharyngeal depth and *A*/*N* ratio. **(A)** correlation analyses between age and adenoid depth, **(B)** correlation analyses between age and ND, **(C)** correlation analyses between age and *A*/*N* ratio, **(D)** correlation analyses between ND and adenoid depth, **(E)** correlation analyses between *A*/*N* ratio and AD, **(F)** correlation analyses between *A*/*N* ratio and ND. AD, adenoid depth; ND, nasopharyngeal depth.

The scatter plot presented in Figure 3A illustrates a weak but statistically significant positive correlation between age and AD (*R*^2^ = 0.01, *P* < 0.001). The regression equation (*Y* = 0.13 × *X* + 13.86) suggests that age-related changes are minimal, with AD increasing by approximately 0.13 mm per year.

#### 3.7.2 Age vs. nasopharyngeal depth

Figure 3B indicates a significant positive correlation (*R*^2^ = 0.34, *P* < 0.001) between age and ND, characterized by an annual increase of 0.80 mm (*Y* = 0.80 × *X* + 18.41). This finding suggests that age accounts for 34% of the variability in nasopharyngeal growth, indicating a more predictable pattern of age-dependent enlargement compared to AD.

#### 3.7.3 Age vs. *A*/*N* ratio

A significant negative correlation was identified between age and the *A*/*N* ratio (*R*^2^ = 0.098, *P* < 0.001). The regression equation (*Y* = −0.015 × *X* + 0.73) indicates an annual decrease of 0.015 in the ratio value, with age explaining 9.8% of the variability in the *A*/*N* ratio.

#### 3.7.4 Adenoid depth vs. nasopharyngeal depth

Figure 3D further reveals a significant positive correlation between AD and ND (*R*^2^ = 0.24, *P* < 0.001), with the regression equation (*Y* = 0.52 × *X* + 15.30) indicating that for every 1 mm increase in ND, AD increases by 0.52 mm.

#### 3.7.5 Adenoid depth vs. *A*/*N* ratio

Figure 3E illustrates a weak yet statistically significant positive correlation between AD and *A*/*N* ratio (*R*^2^ = 0.056, *P* < 0.001). The corresponding regression equation (*Y* = 0.029 × *X* + 0.22) suggests that for each 1 mm increase in AD, the *A*/*N* ratio is expected to increase by 0.029.

#### 3.7.6 Nasopharyngeal depth vs. *A*/*N* ratio

Figure 3F demonstrates a significant negative correlation between ND and *A*/*N* ratio (*R*^2^ = 0.036, *P* < 0.001). The associated regression equation (*Y* = −0.0069 × *X* + 0.80) indicates that for every 1 mm increase in ND, the *A*/*N* ratio decreases by 0.0069.

### 3.8 Comparison of *A*/*N* ratio percentile values by age groups with current clinical classification criteria for adenoid hypertrophy

**Figure 4:**
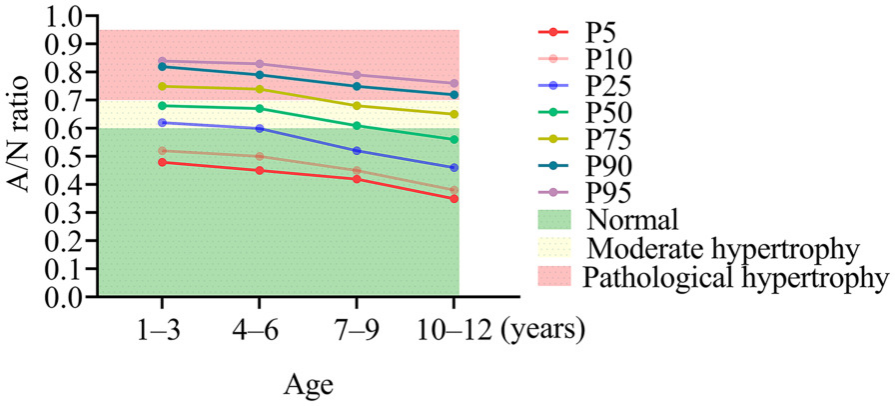
Discrepancies between age-specific *A*/*N* ratio percentiles and conventional diagnostic criteria for adenoid hypertrophy. AD, adenoid depth; ND, nasopharyngeal depth.

Figure 4 highlights the inconsistencies between age-specific *A*/*N* ratio percentiles and the established clinical diagnostic criteria for AH. Utilizing age-adjusted reference ranges (P5–P95), the normal intervals were identified as 0.48–0.84 for ages 1–3 years, 0.45–0.83 for ages 4–6 years, 0.42–0.79 for ages 7–9 years, and 0.35–0.76 for ages 10–12 years. Notably, under the current fixed thresholds, a considerable number of children would be inaccurately classified. For instance, in the 1–3-year age group, the 25th percentile (0.62) exceeded the cutoff for moderate hypertrophy, while the 75th percentile (0.75) reached levels indicative of pathological hypertrophy. Similar patterns were observed in older age groups: among 4–6-year-olds, the median value (0.67) suggested moderate hypertrophy, and the 75th percentile (0.74) indicated pathological hypertrophy. The 7–9-year age group exhibited particularly notable discrepancies, with the median value (0.61) aligning exactly with the threshold for moderate hypertrophy, and the 95th percentile (0.79) signifying pathological enlargement. By the ages of 10–12 years, although the median value (0.56) remained within the normal range, the 75th percentile (0.65) and the 95th percentile (0.76) fell within the moderate and pathological ranges, respectively.

### 3.9 Inter-rater reliability analysis

Tables 4 showed the cross-classification of AH grading by the two radiologists. The inter-rater reliability analysis demonstrated to high consistency between the two radiologists. For categorical classification of AH, the simple agreement was 84.2%. The kappa with intra-rater reliabilities for the two radiologists was kappa = 0.76; 95% CI (0.74–0.78) (Table 5).

**Table 4:** Cross-classification of adenoid hypertrophy grading between two radiologists (*n* = 2,629).

Radiologist A classification	Rater B classification			
	Normal (≤0.60)	Moderate (0.61–0.70)	Pathological (≥0.71)	Total
Normal (≤0.60)	648	83	9	740
Moderate (0.61–0.70)	72	798	132	1,002
Pathological (≥0.71)	11	108	768	887
Total	731	989	909	2,629

**Table 5:** Inter-rater agreement for adenoid hypertrophy (*A*/*N* ratio) classification (*n* = 2,629).

Agreement metric	Value	*P*-value
Simple agreement	84.2%	—
Kappa	0.76 (95% CI, 0.73–0.78)	<0.001

*A*/*N*, adenoid-to-nasopharyngeal.

## 4 Discussion

This extensive retrospective study provides significant insights into the age-specific development of the adenoids and nasopharynx in children, establishing percentile-based reference ranges for AD, ND, and the *A*/*N* ratio across four pediatric age groups (1–12 years). Our findings underscore significant limitations in the current fixed diagnostic criteria for AH, emphasizing the necessity of age-stratified thresholds for accurate clinical assessment.

AH is a prevalent condition affecting approximately 35%–70% of pediatric populations worldwide, positioning it among the most common otolaryngological disorders in children (1). A study conducted in Istanbul, Turkey, which employed portable telescope examination, revealed age-specific prevalence rates of AH: 27% in children aged 5–7 years, decreasing to 19.5% in the 8–10 year age group, and 19.9% among those aged 11–14 years (11). Utilizing an *A*/*N* ratio greater than 0.62 as the diagnostic criterion, the prevalence of AH in 12-year-old children was found to be 17.6% (12). Nasal endoscopy is considered the gold standard for diagnosing AH, as it allows for direct visualization of adenoid size and morphology within the nasopharynx (13, 14). However, due to the challenges associated with obtaining cooperation from pediatric patients and the potential discomfort associated with the procedure, lateral nasopharyngeal radiography serves as an excellent non-invasive alternative (5, 15). Research has established a significant correlation between the *A*/*N* ratio and endoscopic findings in the diagnosis of AH (16). In our study, the prevalence rates of AH (both moderate and pathological) diagnosed via the *A*/*N* ratio demonstrated a clear age-dependent trend: 79.4% in the 1–3 years age group, 74.5% in the 4–6 years group, 54.3% in the 7–9 years group, and 39.8% in the 10–12 years group (Table 1). This observation suggests a gradual decline correlated with advancing age. Nonetheless, the notably high prevalence rates (79.4% in the 1–3 years age group and 74.5% in the 4–6 years age group) raise concerns about the potential overestimation of AH when employing the *A*/*N* ratio criteria, particularly among preschool-aged children. The growth and development of adenoids in children represent a multifaceted process that typically commences shortly after birth, characterized by a period of rapid expansion during early childhood. Peak adenoid development is generally observed between the ages of 3 and 7 years (17). During this developmental phase, adenoids attain their maximum size and functional capacity, which is intricately associated with the maturation of the child's immune system (18). Our findings from lateral nasopharyngeal radiography indicate that peak adenoid development occurs between the ages of 4 and 6 years, followed by a modest increase from ages 7–12 years (Table 1). Correlation analysis further revealed a weak positive association between age and adenoid size (Figure 1A). Notably, in contrast to the relatively gradual growth rate of adenoids, the nasopharyngeal cavity expands at a more accelerated rate with advancing age (Table 1). Additional correlation analysis demonstrated that the positive association between age and ND is stronger than that between age and AD (Figure 1B). Previous research has documented a progressive increase in ND from ages 4–5 to 14–15 years (19). It is noteworthy that the nasopharyngeal free airway space in children aged 10–11 years does not diminish, despite an increase in adenoid thickness. This phenomenon can be attributed to the downward displacement of the hard palate, which results in an enlargement of the free airway space as a result of growth (19). This observation may elucidate the inverse relationship between age and the *A*/*N* ratio identified in our study (Figure 1C). Within our study cohort, the median *A*/*N* ratio exhibited a distinct age-dependent decline: 0.68 (range 0.11–0.95) for children aged 1–3 years, 0.67 (0.17–0.94) for those aged 4–6 years, 0.61 (0.16–0.93) for ages 7–9 years, and 0.56 (0.26–0.88) for ages 10–12 years. The differences among these age groups were statistically significant (Table 1), reinforcing the observation that the prevalence of AH decreases with age, in accordance with prior research (11). Additionally, our findings revealed no significant sex-based differences in these developmental changes, which is consistent with previous studies (11). In addition to physiological growth patterns, extrinsic factors such as obesity may significantly influence nasopharyngeal dimensions. Arens et al. demonstrated that obesity contributes to upper airway obstruction through mechanisms such as pharyngeal fat deposition and mechanical compression, with 45% of obese children with obstructive sleep apnea syndrome (OSAS) exhibiting adenotonsillar hypertrophy. Notably, residual obstruction persists in half of these children following adenotonsillectomy, indicating that obesity-related structural changes (e.g., fat infiltration in lateral pharyngeal walls) may independently affect nasopharyngeal development (20). Furthermore, Daar et al. reported that obese children exhibit significantly higher rates of AH (34% compared to 6% in non-obese controls) and tonsillar hypertrophy (16% compared to 4%) (21). In addition to obesity, allergic conditions also play a critical role in modulating nasopharyngeal dimensions. Specifically, allergic rhinitis has been shown to significantly increase the risk of adenotonsillar hypertrophy (odds ratio = 2.95) through Th2-mediated inflammatory pathways (22). This causal relationship is further substantiated by a recent Mendelian randomization study, which indicates that genetic predisposition to allergic diseases—particularly allergic rhinitis (odds ratio [OR] = 1.14) and allergic asthma (OR = 1.12)—significantly increases the risk of chronic adenotonsillar disease, while no reverse association has been identified (23). Although our study provides normative data for the general pediatric population, these comorbid conditions may necessitate careful consideration in the clinical interpretation of radiographic measurements. Future research should focus on elucidating the specific impacts of these factors on adenoid and nasopharyngeal development to enhance the refinement of diagnostic criteria.

Our study significantly contributes to the field by establishing the first comprehensive percentile-based reference ranges (P5–P95) for AD, ND, and *A*/*N* ratio in outpatient children aged 1–12 years, thereby addressing a critical gap in age-stratified diagnostic standards (Table 3) (1, 13). The observed reference ranges indicate dynamic developmental patterns: while AD remains relatively stable (median 14–15 mm across age groups), ND shows a progressive increase (21–27 mm), which correlates with a decline in the *A*/*N* ratio from 0.68 to 0.56. This finding is consistent with a Brazilian study involving 320 participants aged 4–16 years (all nasal breathers without prior adenoidectomy), which reported *A*/*N* ratios of 0.67 ± 0.04 at ages 4–5 years, decreasing to 0.52 ± 0.05 at ages 14–15 years (19). This physiological trajectory highlights the limitations of fixed diagnostic thresholds, which tend to disproportionately classify younger children as pathological (e.g., 42% of 1–3-year-olds vs. 13.7% of 10–12-year-olds met the *A*/*N* ≥ 0.71 criteria) (Table 1). Previous studies have indicated that nasopharyngeal airway restriction is most prevalent during the preschool and early school years, as AD often expands more rapidly than the physiological growth of ND (18). Moreover, the variability in adenoid and nasopharyngeal growth patterns among individuals complicates the establishment of a standardized diagnostic threshold (17). Some children may exhibit accelerated growth of the nasopharyngeal space, resulting in a more significant reduction in the *A*/*N* ratio, while others may demonstrate slower growth rates. This individual variability highlights the necessity for age-specific or growth-adjusted diagnostic criteria that more accurately reflect the dynamic changes occurring in the nasopharyngeal region throughout childhood development (5).

The discordance between age-specific percentiles and fixed diagnostic criteria (Figure 4) highlights significant risks of systematic misclassification. For instance, according to the current thresholds (≥0.61), 79.6% of children aged 1–3 years would be classified as having moderate-to-severe hypertrophy. However, our data indicate that these values fall within the range of normal developmental variation (P25–P75: 0.62–0.75). These findings advocate for the adoption of age-stratified diagnostic criteria, potentially utilizing percentile thresholds (e.g., P95) of the *A*/*N* ratio for the diagnosis of AH. A systematic review by Major et al. identified significant limitations in current diagnostic modalities for AH, noting that no single optimal screening tool is available due to inherent trade-offs between accuracy, accessibility, and safety. Notably, lateral radiography—the most commonly employed method in clinical practice—exhibited inconsistent performance, with sensitivity ranging from 61% to 75% and specificity from 41% to 55% for adenoid size assessment (13). This further corroborates our findings concerning the substantial misclassification risks associated with the application of fixed *A*/*N* ratio thresholds across different age groups. Although Caylakli et al. (16) proposed a significant correlation between the *A*/*N* ratio and endoscopic findings in the diagnosis of AH; however, their study was constrained by a relatively small sample size (*n* = 85) and a broad age range (2–12 years). These methodological limitations highlight the imperative for the establishment of age-stratified diagnostic criteria for *A*/*N* ratios, as advocated in our current research. Additionally, we align with the perspective of Major et al. (13) that a multifaceted diagnostic approach—combining radiographic indices with a thorough clinical history—can effectively mitigate the shortcomings of individual diagnostic methods and enhance overall accuracy in both the identification and exclusion of AH.

The inter-rater reliability analysis demonstrated substantial agreement (Kappa = 0.76; 95% CI: 0.73–0.78) between radiologists in classifying AH severity using *A*/*N* ratios, with a simple agreement rate of 84.2%. This level of agreement aligns with findings from Kolo et al. (24), who reported Kappa values of 0.67–0.82 for subjective assessments of nasopharyngeal radiographs among otolaryngologists and radiologists, further supporting the reproducibility of radiographic evaluations in clinical practice.

This study has several limitations. The single-center retrospective design may introduce selection bias, limiting the generalizability of findings to the broader pediatric population. The absence of nasoendoscopic evaluation prevents direct validation of radiographic measurements, and the lack of a systematic clinical symptom assessment restricts correlation with functional outcomes. Future prospective multicenter studies should incorporate endoscopic evaluation and comprehensive clinical assessments to validate age-specific *A*/*N* ratio thresholds and improve diagnostic accuracy for adenoid hypertrophy across different age groups.

## 5 Conclusions

This study provides evidence-based tools for age-specific adenoid reference ranges, highlighting deficiencies in the existing fixed *A*/*N* ratio thresholds that may lead to misdiagnosis in pediatric populations. The newly established percentile-based ranges (P5-P95) for AD, ND, and *A*/*N* ratio across four age groups (1–3, 4–6, 7–9, and 10–12 years) offer a more accurate diagnostic reference. The findings support the use of these age-specific criteria to enhance AH diagnosis and reduce misclassification risks. Future research should validate these thresholds through multicenter studies and integrate them with comprehensive clinical assessments.

## Data Availability

The original contributions presented in the study are included in the article/Supplementary Material, further inquiries can be directed to the corresponding authors.
